# Vitamin D status among residents of Molidawa Daur Autonomous Banner, Inner Mongolia, North China

**DOI:** 10.15537/smj.2023.44.4.20220780

**Published:** 2023-04

**Authors:** Jingwen Hu, Yunmei Liang, Guiping Wen, Dezhong Chen, Yuanjing Liu, Hongmei Zhang, Xiaosong Qin

**Affiliations:** *From the Department of Laboratory Medicine (Hu, Qin), Shengjing Hospital of China Medical University, and from Department of Laboratory Medicine (Hu, Qin), Liaoning Clinical Research Center for Laboratory Medicine, Shenyang; from the Department of Pediatrics (Liang), Beijing Chaoyang Hospital Affiliated Capital Medical University, Beijing; from the Department of Pediatrics (Wen, Chen, Liu, Zhang), Molidawa Daur Autonomous Banner People’s Hospital, Molidawa Daur Autonomous Banner, Hulunbuir, Inner Mongolia Autonomous Region, China.*

**Keywords:** vitamin D, vitamin D deficiency, age groups, gender, Inner Mongolia

## Abstract

**Objectives:**

To retrospectively analyze the vitamin D (VD) status of residents in northeastern Inner Mongolia and its relationship with the average monthly sunshine hours.

**Methods:**

Serum 25-hydroxyvitamin D[s-25(OH)D] samples from 4982 outpatients (2092 males) in Moli Dawa Daur Autonomous Banner People’s Hospital, Hulunbuir, China from July 2018 to January 2022 were included in this study.

**Results:**

The overall median s-25(OH)D was 53.3 nmol/L, VD deficiency (<30 nmol/L), deficiency (30-50 nmol/L), sufficient (>50-250 nmol/L) and excess (>250 nmol/L) were 16% (796/4982), 30% (1495/4982), 53.4% (2658/4982) and 0.7% (33/4982). There were statistically significant differences in median s-25(OH)D by month, age-groups and gender (*p*<0.001). Low VD status (LVDS, including VD deficiency and insufficiency) in females was 54.6% and males was 33.9%, and the LVDS composition differed significantly by age-group and month (*p*<0.05). The changing trend of the median s-25(OH)D level was similar to the monthly average sunshine hours, with a slight lag.

**Conclusion:**

Nearly half of residents live in LVDS. LVDS is affected by month, gender, and age.

**V**itamin D (VD) is a liposoluble essential vitamin that regulates bone metabolism and the homeostasis of calcium and phosphorus in the body. Vitamin D deficiency can lead to osteoporosis in adults and rickets in children.^1^ Vitamin D is obtained by a combination of dietary pathways (10-20%) and dermal synthesis under the influence of solar ultraviolet B (UVB) sunlight (80–90%).^1^ The amount and quality of sunlight reaching the terrestrial surface is impacted by season and latitude, particularly in the UVB area of the spectrum.^2,3^ According to one study, even when exposed to clear sunlight, no pre-vitamin D is created from November through February (in Boston, MA, USA; 42.2°N).^4^ This ineffectual solar exposure lasts from October to March at higher latitudes, such as 52°N, whereas VD can be generated in the middle of winter at a latitude of 34°N.^4^ Furthermore, lifestyle, gender, and age may all have a role in VD status.

An estimated one billion people worldwide have inadequate VD levels, and poor VD status continues to be a global public health concern.^5^ China, as a large country, has a wide latitude range, ranging from tropical and subtropical regions in the south to chilly temperate zones in the north. The incidence of low VD status (LVDS, including VD deficiency and insufficiency) varies substantially with differences in geographic location, dietary choice, and economic development between urban and rural areas.^6^ Owing to the popularization of children’s health education in China, the LVDS risk among young children and infants has been effectively improved.^7^ However, the issue of excessive VD in children, and LVDS in adolescents, adults, and elders hardly attracted attention. Molidawa Daur Autonomous Banner is an ethnic minority township in Inner Mongolia, north China, located at high latitude (48.48°N). Locally, adolescents with abnormal spinal development and adults with skeletal deformities of lower extremities are common. Serum 25-hydroxy-vitamin D [s-25(OH)D] is the most common circulating form of VD in the blood and is largely considered the greatest biomarker of VD status.^8,9^ Based on local single-center s-25(OH)D data, this study investigated the effects of age, gender, and month on the status of VD in Molidawa Daur Autonomous Banner. Our results provide a theoretical basis for local prevention and control of LVDS and avoidance of VD overdose.

## Methods

We analyzed retrospective cross-sectional data on the local status of VD. In this study, 4982 blood samples (2092 males) were collected from outpatients aged 0–82 years in the Molidawa Daur Autonomous Banner People’s Hospital who voluntarily visited the hospital during the period from July 2018 to January 2022. The s-25(OH)D data were divided into 4 grades (deficiency, insufficiency, adequacy, and excess), 7 groups by age (0-3, 4-6, 7-11, 12-17, 18-30, 31-50, and 51-82 years old), 2 groups by gender (male and female), and 12 groups by month (from January to December). Ages 0-3, 4-6, 7-11, 12-17, 18-30, 31-50, and 51-82 years were early-stage childhood, late-stage childhood, early-stage adolescence, late-stage adolescence, early-stage adulthood, late-stage adulthood, and elders, respectively. Minors were those under the age of 18. Relationships between the levels of s-25(OH)D levels, age group, gender, and month, as well as the relationship between median monthly s-25(OH)D level and monthly sunshine hours (h) were analyzed. Incomplete data (for example, missing a full date of birth or gender) were excluded from the final analysis. Our research was approved by the local Ethics Committee of Molidawa Daur Autonomous Banner People’s Hospital.

All participants were calm and fasting when blood samples were taken in the morning. Blood was drawn from the anterior elbow vein into serum tubes and centrifuged at 3000 rpm for 5 minutes (min) at ambient temperature. The s-25(OH)D levels were determined within 8 hours after blood isolation by electrochemiluminescence using a Roche Diagnostics Modular Cobas e411 device (Roche Diagnostics, Mannheim, Germany) at Molidawa Daur Autonomous Banner People’s Hospital, Inner Mongolia, China.

There is still no agreement on the cut-off values of s-25(OH)D to set the VD status. We stratified all the participants using the generally accepted thresholds for VD deficiency (s-25(OH)D <30 nmol/L), insufficiency (s-25(OH)D 30-50 nmol/L), adequacy (s-25(OH)D >50–250 nmol/L), and excess (s-25(OH)D >250 nmol/L).^10-12^ The LVDS is referred to as s-25(OH)D ≤50 nmol/L.^10^

### Statistical analysis

All data were analyzed with the Statistical Package fot the Social Sicences for windows, version 22.0 (IBM Corporation, Armonk, NY, USA). Data have been split into separate groups according to the various hypothetical predictors for the VD level as mentioned earlier. The normality of the distribution of all continuous variables was verified through the Kolmogorov–Smirnov test. Continuous variables were depicted with the median because they were inconsistent with the normal distribution. Categorical variables were presented in terms of percentages (%). Group rates were compared through a nonparametric assay. If a comparison was made between 2 independent samples were compared, the Mann–Whitney U test was adopted; if the number of independent samples was greater than 2, the Kruskal–Wallis H test was applicable. If the frequency was <5 or the cases were fewer than 40, Fisher’s exact test was properly utilized. Two-sided *p*-values of <0.05 were applied to determine if the difference was statistically significant.

## Results

### S-25(OH)D levels in Molidawa Daur Autonomous Banner

The median level of s-25(OH)D in 4982 samples in this study was 53.3 nmol/L; the difference between genders was statistically significant (*p*<0.001). Refer to Table 1 for more information.

**Table 1:** - Effect of gender on serum vitamin D level of residents in Molidawa Daur Autonomous Banner.

Gender	n (%)	Median (nmol/L)	Mann-Whitney test		
			U	Z	*P*-value
Male	2092 (42)	63.5	2243605	-15.55	<0.001
Female	2890 (58)	46.95			
Total	4982 (100)	53.3			

Median levels of s-25(OH)D levels among children aged 0-3 years (98.2 nmol/L) and 4-6 years (59 nmol/L) exceeded 50 nmol/L. The median s-25 (OH)D level was less than 50 nmol/L for individuals 7 years and older. Among them, the early-stage adulthood group (aged 18-30 years) had the lowest median level of s-25(OH)D (36.2 nmol/L). A statistically significant difference in the median s-25(OH)D was observed between the various age groups (*p*<0.001). The difference was statistically significant in VD status among minors, adults, and elders (*p*<0.001), being worst for adults and best for minors. See Table 2 for details.

**Table 2:** - Effect of age on serum vitamin D level of residents in Molidawa Daur Autonomous Banner.

Age group	n (%)	Median (nmol/L)	Kruskal-Wallis test	
			H	*P*-value
0~3 years	1535 (30.8)	98.2^*,†, ‡, §,**,‡‡^	2274.8	<0.001
4~6 years^‡‡^	700 (14.05)	59.0^*,†, ‡, §,**,‡‡^		
7~11 years^**^	963 (19.3)	46.0^*,†, ‡,**^		
12~17 years^†^	430 (8.6)	36.7^†^		
18~30 years^*^	790 (15.9)	36.2^*,†,‡,**^		
31~50 years^‡^	464 (9.3)	37.9^‡^		
51~82 years^§^	100 (2.0)	44.3^*,§^		
Minors	3628 (72.8)	63.5	875.3	<0.001
Adults^††^	1254 (25.2)	36.7		
Elders^§^	100 (2.0)	44.3^*,§^		

The labels *,†,‡, §,**,††, and ‡‡ indicate that the distribution of median serum vitamin D in different age groups was significantly different after Bonferroni correction (*p*<0.05/21). The labels § and †† indicate that the distribution of median serum vitamin D in minors, adults, and elders was significantly different after Bonferroni correction (*p*<0.05/3).

The median s-25(OH)D levels of residents did not reach 50 nmol/L from January to May. The median s-25(OH)D level was lowest in February (39.7 nmol/L), began to rise in June, peaked in September (66.3 nmol/L), and then began to decline. There was a statistical difference in the median levels of s-25(OH)D among different months (*p*<0.001). See Table 3 for additional information.

**Table 3:** - Effect of month on serum vitamin D level of residents in Molidawa Daur Autonomous Banner.

Month	n (%)	Median (nmol/L)	Kruskal-Wallis test	
			H	*P*-value
January^¥^	379 (7.6)	45.25^¥^	354.9	<0.001
February*	188 (3.8)	39.7*		
March^‡^	411 (8.3)	43.6^‡^		
April^§^	365 (7.3)	43.7^§^		
May^†^	460 (9.2)	41.5^†^		
June^††^	401 (8.1)	54.15^*,†, ‡,§,††^		
July	498 (10.0)	60.6^*,†, ‡,§¥,**^		
August	585 (11.7)	66.3^*,†,‡,§,¥,**,‡‡,§§^		
September	377 (7.6)	66.3^*,†,‡,§,¥,**,‡‡,§§^		
October^‡‡^	432 (8.7)	55.05^*,†, ‡,§,¥, ‡‡^		
November	453 (9.1)	56.4^*,†,‡,§,¥,§§^		
December**	433 (8.7)	51.1^*,†,‡,§,**^		

The labels *,†, ‡, §, ¥, **, ††, ‡‡, and §§ indicate that the distribution of median serum vitamin D in different months was significantly different after Bonferroni correction (*p*<0.05/66).

**Figure 1:**
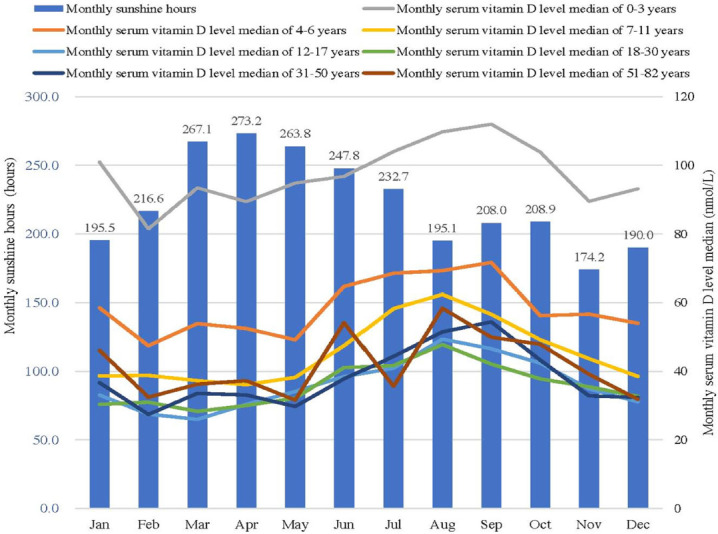
- Relationship between monthly serum vitamin D level median and monthly sunshine hours.

According to the government work report of the weather bureau in Molidawa Daur Autonomous Banner, the average monthly sunshine hours from 2018 to 2021 ranged from 174.2 h in November to 273.2 h in April. Trends in median levels of s-25(OH)D were similar across age groups from January to December (Figure 1).

### The status of VD deficiency, insufficiency, adequacy, or excess in Molidawa Daur Autonomous Banner

As a whole, LVDS accounted for 46% of local residents, and VD excess accounted for 0.7%. The composition of LVDS varied in different age groups. The proportion of LVDS varied by age group, with the lowest in early childhood, in children aged 0-3 years (9.2%), and the highest in late-stage adolescents aged 12-17 years (78.3%) and early-stage adults aged 18-30 years (78.8%). The proportion of LVDS was 35.1% in minors, 76.2% in adults, and 63% in elders. Vitamin D excess occurred in early-stage childhood, late-stage childhood, early-stage adolescents, and late-stage adults, with proportions of 1.6%, 0.9%, 0.2%, and 0.2%, respectively. The percentages of LVDS in females was 54.6% and males was 33.9%, and the VD excess percentage was 0.8% for female and 0.5% for male. The LVDS composition of different genders significantly differed in different age groups (Table 4). The proportion of LVDS varied by month, with the highest in February (65.9%) and the lowest in August (26.7%) and September (28.3%). Vitamin D excess occurred from January to November, with proportions of 0.5%, 1.1%, 0.7%, 1.4%, 0.9%, 0.2%, 0.6%, 0.5%, 0.8%, 1.2%, and 0.4%, respectively. Except for January and February, the LVDS composition for different genders significantly differed in different months (*p*<0.001) (Table 5).

**Table 4:** - Serum vitamin D status varies by gender in different age groups.

Age groups	Gender	Total number	Deficiency <30 nmol/L	Insufficiency 30-50 nmol/L	Adequacy >50-250 nmol/L	Excess >250 nmol/L	Mann-Whitney test		
							U	Z	*P*-value
0~3 years	Male	824	26 (3.2)	54 (6.6)	736 (89.3)	8 (1.0)	286279.0	-1.4	0.153
	Female	711	20 (2.8)	41 (5.8)	634 (89.2)	16 (2.3)			
4~6 years	Male	388	12 (3.1)	102 (26.3)	273 (70.4)	1 (0.3)	54370.0	-2.8	0.005
	Female	312	28 (9.0)	94 (30.1)	185 (59.3)	5 (1.6)			
7~11 years	Male	560	68 (12.1)	224 (40.0)	266 (47.5)	2 (0.4)	96615.0	-4.2	<0.001
	Female	403	65 (16.1)	201 (49.9)	137 (34.0)	0 (0.0)			
12~17 years	Male	270	66 (24.4)	134 (49.6)	70 (25.9)	0 (0.0)	16326.0	-4.6	<0.001
	Female	160	73 (45.6)	64 (40.0)	23 (14.4)	0 (0.0)			
18~30 years	Male	7	1 (14.3)	3 (42.9)	3 (42.9)	0 (0.0)	1930.5	-1.45	0.182
	Female	783	261 (33.3)	357 (45.6)	165 (21.1)	0 (0.0)			
31~50 years	Male	24	4 (16.7)	9 (37.5)	11 (45.8)	0 (0.0)	3934.5	-2.2	0.029
	Female	440	154 (35.0)	167 (38.0)	118 (26.8)	1 (0.2%			
51~82 years	Male	19	2 (10.5)	5 (26.3)	12 (63.2)	0 (0.0)	521.0	-2.4	0.016
	Female	81	16 (19.8)	40 (49.4)	25 (30.9)	0 (0.0)			
Total		4982	796(16.0)	1495(30.0)	2658(53.4)	33(0.7)			

Values are presented as number and percentages (%).

**Table 5:** - Serum vitamin D status varies by gender in different months.

Month	Gender	Total number	Deficiency <30 nmol/L	Insufficiency 30-50 nmol/L	Adequacy >50-250 nmol/L	Excess >250 nmol/L	Mann-Whitney test		
							U	Z	*P*-value
January	Male	190	29 (15.3)	74 (38.9)	87 (45.8)	0 (0.0)	16455.0	-1.5	0.132
	Female	189	51 (27.0)	57 (30.2)	79 (41.8)	2 (1.1)			
February	Male	80	23 (28.8)	23 (28.8)	34 (42.5)	0 (0.0)	3695.0	-1.8	0.075
	Female	108	40 (37.0)	38 (35.2)	28 (25.9)	2 (1.9)			
March	Male	162	29 (17.9)	53 (32.7)	80 (49.4)	0 (0.0)	15866.5	-3.9	<0.001
	Female	249	86 (34.5)	81 (32.5)	79 (31.7)	3 (1.2)			
April	Male	148	22 (14.9)	55 (37.2)	69 (46.6)	2 (1.4)	12779.5	-3.5	<0.001
	Female	217	69 (31.8)	74 (34.1)	71 (32.7)	3 (1.4)			
May	Male	156	21 (13.5)	53 (34.0)	82 (52.6)	0 (0.0)	16948.5	-5.3	<0.001
	Female	304	99 (32.6)	116 (38.2)	85 (28.0)	4 (1.3)			
June	Male	153	7 (4.6)	38 (24.8)	108 (70.6)	0 (0.0)	14008.0	-4.9	<0.001
	Female	248	46 (18.5)	85 (34.3)	116 (46.8)	1 (0.4)			
July	Male	213	5 (2.3)	41 (19.2)	165 (77.5)	2 (0.9)	22591.0	-5.8	<0.001
	Female	285	36 (12.6)	93 (32.6)	155 (54.4)	1 (0.4)			
August	Male	294	4 (1.4)	43 (14.6)	246 (83.7)	1 (0.3)	33637.5	-5.8	<0.001
	Female	291	14 (4.8)	95 (32.6)	180 (61.9)	2 (0.7)			
September	Male	159	4 (2.5)	24 (15.1)	131 (82.0)	0 (0.0)	14247.0	-3.7	<0.001
	Female	218	15 (6.9)	64 (29.4)	136 (62.4)	3 (1.4)			
October	Male	162	6 (3.7)	31 (19.1)	121 (74.7)	4 (2.5)	15009.5	-6.2	<0.001
	Female	270	44 (16.3)	95 (35.2)	130 (48.1)	1 (0.4)			
November	Male	206	10 (4.9)	51 (24.8)	143 (69.4)	2 (1.0)	17985.5	-6.0	<0.001
	Female	247	54 (21.9)	81 (32.8)	112 (45.3)	0 (0.0)			
December	Male	169	19 (11.2)	45 (26.6)	105 (62.1)	0 (0.0)	17640.0	-4.0	<0.001
	Female	264	63 (23.9)	85 (32.2)	116 (43.9)	0 (0.0)			

Values are presented as number and percentages (%).

## Discussion

Low VD status is strongly associated with osteomalacia, bone loss and fractures, including pediatric nutritional rickets, osteoporosis and osteomalacia in adults, and hip fractures in elders, and remains prevalent in both developing and developed countries.^9,13,14^ Although there is no uniform standard for the optimal level of VD in the human body, it is generally accepted that concentrations of s-25(OH)D levels under 30 nmol/L raise the risk of osteoporosis or rickets, and that in the range of 50 to 250 nmol/L appears to be secure and sufficient in the overall population for the health of the skeletal health.^11^ This retrospective analysis showed that nearly half of the residents of the Molidawa Daur Autonomous Banner were in LVDS according to the above criteria. The VD status of minors was better than that of adults and elders. Similar to the results of a Korean study, adults were the group at highest risk for LVDS.^15^ As society ages, bone health issues in adults and elders deserve more attention.

The data suggested that median s-25(OH)D levels were best in early-stage childhood, lower in late-stage childhood and early-stage adolescence, lowest in late-stage adolescence and adulthood, and slightly improved in elders. These data confirmed that the median s-25(OH)D levels were closely related to age and similar to previous findings (in the US, UK and Germany).^16^ The incidence of LVDS was highest among late-stage adolescents and early-stage adulthood in the Molidawa Daur Autonomous Banner, China. This is similar to situation in Europe, the Duhok area in Iraq, and Sichuan, China.^16-18^ Compared with previous studies in northern China at a similar latitude (44.04-46.40°N), minors in the Molidawa Daur Autonomous Banner had similar levels of s-25(OH)D and age-related patterns among minors in Heilongjiang Province, but a lower percentage of LVDS, which was related to different criteria for diagnosing LVDS.^7^ Compared with residents in Sichuan Province (26.03–34.19 °N) in China and in Greece (39.07 °N), adults in Molidawa Daur Autonomous Region had lower s-25(OH)D levels.^18,19^ Why this difference can be related to the hours of sunshine or solar UVB radiation in the three regions.

The major source of VD for humans is dermal synthesis. Ultraviolet B-rich sunlight is a critical environmental factor in the dermal transformation of VD.^7^ The sunlight exposure time (sunshine hours) is a measure of the amount of UVB that varies by season and geographic location (latitude).^16^ The geographic location (latitude) and season can therefore affect people’s VD status.^20^ The synthesis of VD in the skin is not considered to be effective in regions above 35°N latitude (including Poland; 49–54°N latitude).^21^ However, it has been reported that dermal VD can be produced at high latitudes of 70°N.^22^ Molidawa Daur Autonomous Banner had the most sunshine hours in April (late spring) and the least sunshine hours in November (early winter). The median s-25(OH)D levels were at LVDS levels from January to May (late winter and early summer), lowest in February (early spring), began to climb in June (summer), and peaked in August–September (autumn), slightly decreasing from October–December (winter), to a moderate state. The percentage of residents with LVDS was highest in February and lowest in August–September. These data suggest that the VD status of residents in the Molidawa Daur Autonomous Banner (48.48°N) is influenced by month and solar UVB light exposure. Trends in median s-25(OH)D levels and monthly sunshine hours were similar, but with a lag. This lag was more pronounced than in a European study.^23^ The reason for this lag is related to 2 factors, one being the local temperature change and the other the physiological process of VD synthesis in the skin.^24^ Although there are more sunshine hours from March to June each year, the average temperature from January to May was below 15°C, and the area of skin exposed to the solar UVB light was far less than that from June to September. This shows that the month of the year can change people’s lifestyle through temperature fluctuations, which in turn affects the production of VD from solar UVB light.

The physiological input of VD comes either from food or from endogenous synthesis of the skin exposed to UVB rays, but the primary source of VD is from its photochemical synthesis in skin through the action of solar UVB rays.^21^ The intake of VD in food as a natural nutrient is incapable of maintaining a good vitamin D condition. In regions where the solar UVB light is insufficient, supplementing VD has become an important complementary means of correcting LVDS. The accumulation of VD supply through skin exposure to solar UVB light rarely causes hypervitaminosis D, whereas excessive intake of VD is potentially toxic.^24,25^ Currently, the dose of VD supplementation in LVDS patients varies from region to region.^26,27^ With the usage of VD supplementation, incidents of VDI (VDI: also called VD toxicity or hypervitaminosis D) are inevitable.^28^ VDI is uncommon, but early diagnosis is required to avoid severe acute and long-term complications, such as hypercalcemia, hypercalciuria, nephrocalcification, nephrolithiasis and renal dysfunction, and even mortal situations.^29,30^ Events of VDI can occur in every age group.^31-33^ However, the VDI criterion for s-25(OH)D is controversial.^26,32,34^ In this report, the therapeutic dose of VD supplementation in Molidawa Daur Autonomous Banner was 2,000 IU/day (50 µg) for 3 months, and s-25(OH)D > 250nmol/L was identified as VDI.^25,35^ Our data showed that individuals with s-25(OH)D levels above 250 nmol/L were found among minors below 11 years old and adults aged 31-50. The incidence was higher in minors than in adults (one case), and the highest incidence was in young children. Except for December, there were cases of children with s-25(OH)D levels exceeding 250 nmol/L every month. Although VD excess rates were highest in April, there was no regularity. This result indicated that the s-25(OH)D >250 nmol/L status of local residents was not caused by month (season), but was caused by excessive intake of VD supplementation, and confirmed that the dose of 2000 IU/day of VD supplementation for a 3-month course was relatively excessive for children, especially young children.^36^ Therefore, in the treatment of LVDS, factors such as month and age should be considered, and s-25(OH)D should be supervised to avoid the occurrence of VDI.

Gender is a notable factor affecting VD status.^20^ The data in this report show that, overall, the median s-25(OH)D in females was considerably lower than that in males, and females had significantly higher percentages of LVDS. This conclusion is consistent with research findings in many regions.^9,17^ These data also showed that VD status in early childhood was not affected by gender and was similar to that of Greek counterparts.^37^ Vitamin D status in late-stage children and adolescents was influenced by gender, which differed from the findings of European and Greek counterparts.^9,37^ These differences in VD status are thought to be related to supplemental VD intake and lifestyle (outdoor activities). When compared with other age groups, the median s-25(OH)D in early-stage childhood was higher, and the seasonal variation was not obvious. This may be caused by the intake of VD supplementation in early-stage childhood under the guidance of local doctors in accordance with the “Practice Guidelines for Clinical Issues Related to Vitamin D Nutrition in Children in China”, which attenuated the effect of gender factors on VD status.^38^ In addition, outdoor activities in early-stage childhood are rarely influenced by gender. These data also showed that the VD status in early-stage adulthood (aged 18-30) was also not affected by gender, which is consistent with the view of Romanian researchers.^39^ This may be related to the exuberance of female hormones in early-stage adulthood.^40^ However, it may also be related to the bias caused by the unbalanced sample size of males and females. At the same time, it was also shown that the VD status of residents was not affected by gender in January-February. This may be related to changes in people’s lifestyles caused by changes in temperature. Given that January–February is the coldest season in north China (the local temperature fluctuates around -30°C), whether male or female, residents of any age group undertake significantly less outdoor activity, and they wear more clothes during outdoor activities. Their skin is barely exposed to UVB light.

In conclusion, nearly half of residents live in LVDS. Low VD status is affected by month, gender, and age. median s-25(OH)D levels were best in early-stage childhood, lower in late-stage childhood and early-stage adolescence, lowest in late-stage adolescence and adulthood, and slightly improved in elders. The median s-25(OH)D levels were at LVDS levels from January to May (late winter and early summer), lowest in February (early spring), began to climb in June (summer), and peaked in August–September (autumn), slightly decreasing from October–December (winter), the median s-25(OH)D in females was considerably lower than that in males, and females had significantly higher percentages of LVDS.

The disadvantage of this study was that, in addition to age, gender, and month (season), factors such as smoking, genetics, physiological status, obesity, skin color, and the use of cosmetics also affect VD synthesis, but this report did not carried relevant analysis.
